# Methods of induction of labour: a systematic review

**DOI:** 10.1186/1471-2393-11-84

**Published:** 2011-10-27

**Authors:** Ellen L Mozurkewich, Julie L Chilimigras, Deborah R Berman, Uma C Perni, Vivian C Romero, Valerie J King, Kristie L Keeton

**Affiliations:** 1 Department of Obstetrics and Gynecology, Division of Maternal-Fetal Medicine, University of Michigan, 1500 E. Medical Center Drive, Ann Arbor, MI 48109-0264, USA; 2 Department of Family Medicine, Oregon Health & Science University, 3181 SW Sam Jackson Park Road, Portland, OR., 97239-7591, USA; 3 Maternal-Fetal Medicine, Department of Obstetrics and Gynecology, Integrated Health Associates, 24 Frank Lloyd Wright Drive, Ann Arbor, MI, 48105, USA

## Abstract

**Background:**

Rates of labour induction are increasing. We conducted this systematic review to assess the evidence supporting use of each method of labour induction.

**Methods:**

We listed methods of labour induction then reviewed the evidence supporting each. We searched MEDLINE and the Cochrane Library between 1980 and November 2010 using multiple terms and combinations, including labor, induced/or induction of labor, prostaglandin or prostaglandins, misoprostol, Cytotec, 16,16,-dimethylprostaglandin E2 or E2, dinoprostone; Prepidil, Cervidil, Dinoprost, Carboprost or hemabate; prostin, oxytocin, misoprostol, membrane sweeping or membrane stripping, amniotomy, balloon catheter or Foley catheter, hygroscopic dilators, laminaria, dilapan, saline injection, nipple stimulation, intercourse, acupuncture, castor oil, herbs. We performed a best evidence review of the literature supporting each method. We identified 2048 abstracts and reviewed 283 full text articles. We preferentially included high quality systematic reviews or large randomised trials. Where no such studies existed, we included the best evidence available from smaller randomised or quasi-randomised trials.

**Results:**

We included 46 full text articles. We assigned a quality rating to each included article and a strength of evidence rating to each body of literature. Prostaglandin E2 (PGE2) and vaginal misoprostol were more effective than oxytocin in bringing about vaginal delivery within 24 hours but were associated with more uterine hyperstimulation. Mechanical methods reduced uterine hyperstimulation compared with PGE2 and misoprostol, but increased maternal and neonatal infectious morbidity compared with other methods. Membrane sweeping reduced post-term gestations. Most included studies were too small to evaluate risk for rare adverse outcomes.

**Conclusions:**

Research is needed to determine benefits and harms of many induction methods.

## Background

The incidence of labour induction has increased over the last decade [1]. Labour induction may be indicated by medical or obstetrical complications of pregnancy or may be requested or chosen for non-medical or social reasons. When a woman and her care provider decide that labor induction is desired, they must next choose a method of induction. Several factors may influence the choice of method for induction of labour including cervical and membrane status, parity, and patient and provider preference. In this paper we review the evidence for effectiveness of pharmacologic, mechanical, investigational, and complementary and alternative medicine means of third trimester labour induction. We also address possible harms of each method.

We conducted this review to summarize the best evidence available for pregnant women requiring induction of labor in the third trimester of pregnancy with a live fetus. We compared each method with placebo and with other methods of labor induction. The outcomes of this review were the clinically important benefits and harms of labor induction specified by the Cochrane Collaboration's Pregnancy and Childbirth Group in their generic protocol for induction of labour [2].

## Methods

We conducted a comprehensive literature search of the English language literature using Medline and the Cochrane Database of Systematic Reviews. The search covered the period from January 1980 to November 2010. We used combinations of the following search terms "labor, induced/or induction of labor; prostaglandin or prostaglandins, misoprostol; Cytotec; 16,16,-dimethylprostaglandin E2 or E2; dinoprostone; Prepidil; Cervidil: Dinoprost; Carboprost or hemabate; prostin, oxytocin, misoprostol, prostaglandins, membrane sweeping or membrane stripping, amniotomy, balloon catheter or Foley catheter, hygroscopic dilators, laminaria, dilapan, saline injection, nipple stimulation, intercourse, acupuncture, castor oil, herbs". Titles and abstracts were reviewed for possible exclusion by two reviewers (KK or EM and JC). If both reviewers excluded a citation, we eliminated that publication from further review. If at least one reviewer felt the citation might be included or if there was insufficient information to make a determination from the title and abstract, we obtained the full article for review. We identified additional articles for consideration of inclusion through cross checks of relevant bibliographies. Reference lists were created and full-text articles were retrieved for further consideration for inclusion.

In accordance with published guidelines for a "best evidence" review,[3-5] this study included high-quality systematic reviews and randomised controlled trials in a hierarchical fashion. If a high-quality systematic review was available, only randomised controlled trials (RCT) published after the search date for the systematic review were included, except in the instance in which we found a RCT that had not been identified by the systematic review's search or a RCT that had been identified by the systematic review's search but which was awaiting classification. In addition, we included studies with at least one other comparison group (control, placebo or another method) for women undergoing induction of labour at term with a live fetus. We excluded systematic reviews dealing exclusively with subgroups of participants, such as nulliparas or women with prelabour rupture of membranes or with only a particular dose or formulation of the method under study (i.e. low dose or sustained-release preparations). We excluded dose-ranging studies, comparisons of two different formulations of the same method and studies in which subjects in one or more treatment arm received several different methods of labour induction. We did not exclude studies in which subjects received oxytocin augmentation after cervical ripening.

If five or more randomised controlled trials involving a method of induction were published subsequent to the search date of the most recent included systematic review or were "awaiting classification" in the systematic review, we conducted meta-analyses of the primary outcomes reported in these studies. Two authors, VR and UP extracted data independently. Differences were resolved by a third reviewer (EM) after careful review of each manuscript. The new data were added to the data on the comparison available in the Cochrane review. We computed risk ratios and 95% confidence intervals for the main outcome measures reported in these subsequent studies using *Comprehensive Meta-Analysis*, Version 2, Englewood, NJ. We used the fixed effects method for these analyses in order to match the measures of effect reported by the included Cochrane reviews.

We arranged the methods of labour induction according to types including pharmacologic methods, non-pharmacologic methods, complementary and alternative medicine methods, and investigational methods. However, for comparisons of methods with each other, we followed the pre-specified hierarchy used for the series of induction of labour Cochrane Reviews and arranged labour induction methods in that specific order [2]. In each subsection of this paper, we compare each method with those methods prior to it on this list. (see Table 1)

**Table 1:** Induction of labour methods; hierarchy of comparisons[2]

(1)	placebo/no treatment
(2)	vaginal prostaglandin E2
(3)	intracervical prostaglandin E2
(4)	intravenous oxytocin
(5)	amniotomy
(6)	intravenous oxytocin with amniotomy
(7)	vaginal misoprostol
(8)	oral misoprostol
(9)	mechanical methods including extra-amniotic Foley catheter
(10)	membrane sweeping
(11)	extra-amniotic prostaglandins
(12)	intravenous prostaglandins
(13)	oral prostaglandins, excluding misoprostol
(14)	mifepristone
(15)	oestrogens with or without amniotomy
(16)	corticosteroids
(17)	relaxin
(18)	hyaluronidase
(19)	castor oil, bath, and/or enema
(20)	acupuncture
(21)	breast stimulation
(22)	sexual intercourse
(23)	homoeopathic methods
(24)	isosorbide mononitrate
(25)	buccal or sublingual misoprostol
(26)	hypnotic relaxation

All full text articles were independently reviewed by two authors (EM and KK) for possible inclusion. In order to be included in this review, trials had to report on one or more of the outcomes of interest specified by the Cochrane Collaboration induction of labour generic protocol [2]. The Cochrane generic protocol identified the most clinically important benefits and harms of labor induction as the outcomes of interest. These included the following five primary outcomes which were felt to be of most clinical importance: vaginal delivery not achieved within 24 hours (or period specified by authors), uterine hyperstimulation with fetal heart rate (FHR) changes, caesarean section, serious neonatal morbidity or perinatal death (e.g. seizures, birth asphyxia defined by trialists, neonatal encephalopathy, disability in childhood), serious maternal morbidity or death (e.g. uterine rupture, admission to intensive care unit, septicaemia) [2].

Secondary outcomes included unfavourable or unchanged cervix after 12 or 24 hours, need for oxytocin augmentation, uterine hyperstimulation without FHR changes, uterine rupture, epidural analgesia, instrumental vaginal delivery, meconium stained amniotic fluid, Apgar score less than seven at five minutes, neonatal intensive care admission, neonatal encephalopathy, perinatal death, disability in childhood, maternal side effects including nausea, vomiting and diarrhea. Other secondary outcomes included postpartum hemorrhage, serious maternal complications, maternal infections including chorioamnionitis and endometritis, and neonatal infections including meningitis, pneumonia, and sepsis. Maternal satisfaction data were included when available. For each of the methods of induction, we reported the significant measures of effect (odds ratios or risk ratios) on our outcomes of interest from the included systematic reviews and RCTs.

Due to the large number of methods, comparisons and outcomes, we did not include discussion of subgroup analyses. However, because of the importance of cervical status as a determinant of failure of induction of labor to achieve vaginal birth, we reported on the effect of induction methods on caesarean deliveries for the subgroup with unfavorable cervices, where available in the Cochrane reviews.

Two authors (EM and JC) assigned quality scores to each included full-text article based on the Scottish Intercollegiate Guidelines Network (SIGN) quality assessment instruments. These quality assessment instruments are designed to assess the internal validity of each study, and the degree to which the studies' performance minimized bias [6]. The Scottish Intercollegiate Guidelines Network publishes methodology checklists for critical appraisal of both randomised controlled trials and for systematic reviews [6].

We systematically reviewed benefits and harms of each induction method and calculated number needed to treat (NNT) and number needed to harm (NNH) for each significant comparison among methods. For comparisons including only one trial, we used the "treat as one trial" method of calculating the NNT [7]. When more than one trial was included in the comparison, we calculated NNT from pooled odds ratios and risk ratios reported in the included meta-analyses using the *Visual Rx, version 2*; this method is less prone to bias than the "treat as one trial" method of NNT calculation [8,9]. For the purpose of NNT calculations from pooled estimates, we used risk ratios or odds ratios where reported for adverse outcomes and odds ratios to calculated NNT from positive outcomes [9]. When odds ratios were not available in the source studies, we calculated them from available data using *Comprehensive Meta-Analysis, Version 2*, Englewood, NJ. NNT estimates were rounded up to the next whole number whereas NNH estimates were rounded down to the nearest whole number [7,10].

For each method of induction, two authors (EM and KK) assigned a level of evidence based on the "GRADE" (Grading of Recommendations Assessment, Development and Evaluation) system [11]. In this system, the overall strength of evidence is assigned not only based on study design and conduct, but also on factors such as the consistency and precision of the results and the likelihood of publication bias. Overall strength of evidence is classified in the GRADE system as high, intermediate, low or very low. The levels of evidence were assigned in the following manner. If the preponderance of evidence supporting a particular method of labor induction for the outcomes of interest is strong enough that further research would be unlikely to change the reviewers' confidence in the estimate of effect, the evidence quality was assessed as high [11]. If further research would be likely to have an important impact on confidence in the estimate, the evidence quality was assessed as moderate [11]. If further research would be very likely to have an important impact in the estimate of effect, the quality of evidence was assessed as low, and if the estimate of effect is very uncertain, the evidence was assessed as very low [11].

These same authors (EM and KK) also assigned a balance of benefits and harms and a grade of recommendation according to GRADE system guidelines [11,12]. For each clinical intervention under study, the balance of benefits and harms is assessed, and a grade of recommendation is classified as strong or weak. This systematic review does not have a "stand alone" study protocol. In reporting outcomes from included study, we followed PRISMA guidelines [13].

This is a systematic review of previously-published data and as such does not require ethics approval.

## Results

**Figure 1:**
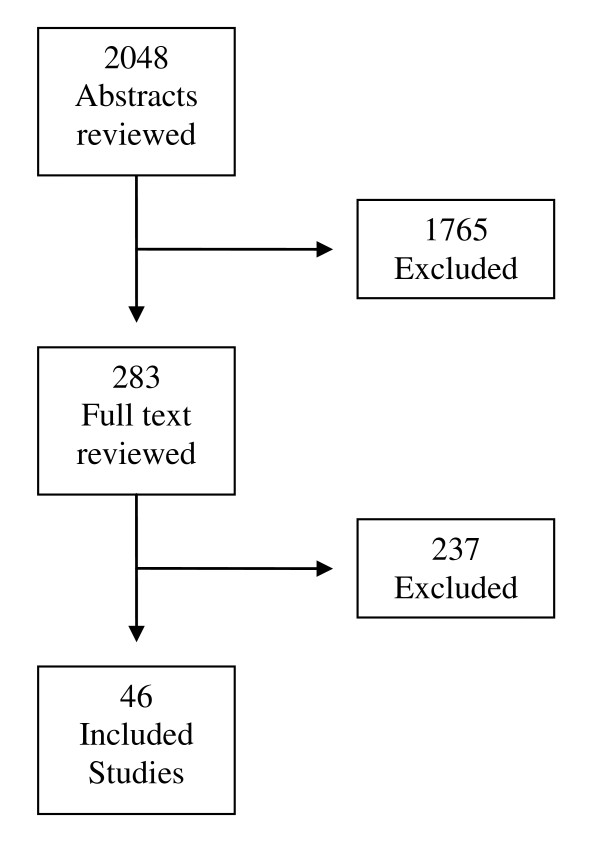
**Flow Diagram**.

We reviewed 2048 abstracts, of which 283 full text articles were examined for further consideration for inclusion and from which 46 studies were included. Thus, we included a total of 46 studies in this systematic review [14-59]. Included studies are listed in Table 2. The flow of abstracts and articles through the review process is outlined in Figure 1. A summary of the overall quality of evidence and strength of recommendation for each intervention is presented in Table 3.

**Table 2:** Included Studies

Author	Year	Indication	Study Design	SR Final Search Date	Study Quality
Kelly[14]	2009	Vaginal Prostaglandins	SR, MA	May 2009	High
Boulvain[15]	2009	Cervical Prostaglandins	SR, MA	August 2007	High
Alfirevic[16]	2010	Intravenous oxytocin	SR, MA	January 2009	High
Kunt[17]	2010	Intravenous oxytocin	RCT		Medium
Bricker[18]	2009	Amniotomy	SR, MA	January 2007	High
Howarth[19]	2009	Intravenous oxytocin plus amniotomy	SR, MA	September 2009	High
Hofmeyr[20]	2010	Vaginal misoprostol	SR, MA	April 2010	High
Alfirevic[21]	2008	Oral misoprostol	SR, MA	May 2008	High
Gaffaney[22]	2009	Oral misoprostol	RCT		High
Nagpal[23]	2009	Oral misoprostol	RCT		High
Muzonzini[24]	2009	Buccal misoprostol	SR, MA	December 2003	High
Bartusevicius[25]	2005	Buccal misoprostol	SR	2004	High
Souza[26]	2008	Buccal misoprostol	SR	February 2008	High
Lo[27]	2006	Buccal misoprostol	RCT		High
Elhassan[28]	2007	Buccal misoprostol	RCT		High
Boulvain[29]	2010	Mechanical methods	SR, MA	April 2001	High
Heinemann[30]	2005	Mechanical methods	SR, MA	November 2005	High
Vaknin[31]	2010	Mechanical methods	SR, MA	April 2008	High
Moraes Filho[32]	2010	Mechanical methods	RCT		High
Boulvain[33]	2009	Membrane sweeping	SR, MA	July 2009	High
Kaul[34]	2004	Membrane sweeping	RCT		High
Kashanian[35]	2006	Membrane sweeping	RCT		High
De Miranda[36]	2006	Membrane sweeping	RCT		High
Hill[37]	2008	Membrane sweeping	RCT		High
Yildirim[38]	2010	Membrane sweeping	RCT		High
Hamdan[39]	2009	Membrane sweeping	RCT		High
Kelly[40]	2009	Castor oil	SR, MA	August 2009	High
Smith[41]	2009	Acupuncture	SR, MA	January 2008	High
Selmer-Olsen[45]	2007	Acupuncture	RCT		High
Smith[42]	2008	Acupuncture	RCT		High
Asher[43]	2009	Acupuncture	RCT		High
Modlock[44]	2010	Acupuncture	RCT		High
Kavanaugh[46]	2009	Breast Stimulation	SR, MA	September 2009	High
Kavanaugh[47]	2009	Sexual Intercourse	SR, MA	June 2007	High
Smith[48]	2010	Homeopathic methods	SR, MA	December 2009	High
Omer[49]	1987	Hypnotic relaxation	Quasi-randomised		Low
Hutton[50]	2009	Extra-amniotic prostaglandins	SR, MA	June 2009	High
Luckas[51]	2010	Intravenous prostaglandins	SR, MA	May 2010	High
French[52]	2009	Oral prostaglandins	SR, MA	July 2009	High
Hapangama[53]	2009	Mifepristone	SR, MA	May 2009	High
Thomas[54]	2008	Oestrogen	SR, MA	January 2008	High
Kavanaugh[55]	2006	Corticosteroids	SR, MA	December 2005	High
Kelly[56]	2009	Relaxin	SR, MA	August 2009	High
Kavanaugh[57]	2009	Hyaluronidase	SR, MA	July 2009	High
Osman[59]	2006	Isosorbide mononitrate	RCT		High
Habib[58]	2008	Isosorbide mononitrate	RCT		High

Abbreviations: SR--Systematic Review; MA--Meta-analysis; RCT--Randomised controlled trial

**Table 3:** Summary: Quality of Evidence and Grades of Recommendation[11,12]

Method	Quality of evidence	Balance of Benefits/Harms	Grade of Recommendation
Vaginal PGE2	Moderate	Trade-offs	Strong
Cervical PGE2	Moderate	Net benefits	Strong
Intravenous oxytocin	Moderate	Trade-offs	Strong
Amniotomy	Moderate	Uncertain trade-offs	Weak
Intravenous oxytocin plus Amniotomy	Moderate	Trade-offs	Strong
Vaginal misoprostol	Moderate	Trade-offs	Strong
Oral misoprostol	Moderate	Trade-offs	Strong
Mechanical methods	Moderate	Trade-offs	Weak
Membrane sweeping	Moderate	Net benefits	Strong
Extra-amniotic prostaglandins	Moderate	No net benefit	Strong (against)
Intravenous prostaglandins	Moderate	Net harms	Strong (against)
Oral prostaglandins	Moderate	Net harms	Strong (against)
Mifepristone	Moderate	Net harms	Weak
Oestrogens	Very Low	Uncertain trade-offs	Weak
Corticosteroids	Very Low	Uncertain trade-offs	Weak
Relaxin	Moderate	Uncertain trade-offs	Weak
Hyaluronidase	Very low	Uncertain trade-offs	Weak
Castor oil	Very Low	Net harms	Strong (against)
Acupuncture	Moderate	No net benefit	Weak
Breast stimulation	Moderate	Uncertain trade-offs	Weak
Sexual intercourse	Very low	Uncertain trade-offs	Weak
Homeopathic Methods	Very low	Uncertain trade-offs	Weak
Isosorbide mononitrate	Moderate	Uncertain trade-offs	Weak
Buccal or sublingual misoprostol	Moderate	Trade-offs	Strong
Hypnosis	Very low	No net benefit	Weak

## Pharmacologic Methods

### Intravaginal prostaglandins (PGE2 and PGF2a)

Our search identified one Cochrane systematic review of vaginal prostaglandin E2 (PGE2) or F2α (PGF2α) [14]. Within this review, 37 studies compared PGE2 with placebo. Of these, two trials with 384 women addressed the primary outcome of achieving vaginal delivery within 24 hours. These studies demonstrated that PGE2 reduced failure to achieve vaginal delivery within 24 hours compared with placebo (36/199 versus 183/185; Relative Risk [RR] 0.19, 95% Confidence Interval [CI] 0.14 to 0.25; NNT = 2). However, there was significant between-study heterogeneity in these two included studies, (*P *< 0.0001), possibly resulting from differences in baseline characteristics of included women and differences in dosing regimens studies [14].

Thirty-four trials with 6399 women compared rates of caesarean section and demonstrated similar rates between PGE2 and placebo groups. Fourteen trials including 1259 women reported that uterine hyperstimulation with FHR changes was increased with vaginal PGE2 compared with placebo (28/642 versus 3/617; RR 4.14, 95% CI 1.93 to 8.90; NNH = 65). Additionally, 13 trials with 3636 women demonstrated that hyperstimulation without FHR changes was also increased (26/1846 versus 7/1790; RR 2.48, 95% CI 1.17 to5.26; NNH = 174). Insufficient data prohibited any conclusions about serious maternal or neonatal morbidity or death [14]. Three trials with 387 women compared PGF2α with placebo. PGF2α reduced the need for oxytocin augmentation (2 trials, 122 women, 41/76 versus 41/46; RR 0.65, 95% CI 0.53 to 0.81, NNT = 4). PGF2α reduced the risk for instrumental vaginal delivery in 2 trials 355 women, 51 of 225 versus 48 of 130, RR 0.60, 95% CI 0.43 to 0.84, NNT = 7) and for epidural analgesia in 3 trials with 387 women (53/241 versus 47 of 146, RR 0.72, 95% CI 0.53 to 0.98, NNT = 17) compared with placebo. These trials did not demonstrate a difference in cesarean section rates or any other outcomes of interest between PGF2α and placebo [14].

#### Unfavorable cervix subgroup

The authors conducted subgroup analyses of trial participants who had cervices unfavorable for induction. There was no difference in the risk of caesarean delivery between vaginal PGE2 and placebo in the subgroup of women with unfavorable cervices (22 trials, 2173 women, 225/1093 versus 254/1080, RR 0.87, 95% CI 0.75 to 1.02) [14].

*Summary: Compared with placebo, vaginal PGE2 increases vaginal delivery rates within 24 hours. However, overall risk of cesarean section was not changed. PGE2 increases uterine hyperstimulation with FHR changes*.

### Cervical PGE2

Our search identified one Cochrane systematic review of the use of intracervical prostaglandins for cervical ripening and induction of labour compared with placebo/no treatment [15]. This Cochrane review included 28 trials with 3764 women that compared intracervical PGE2 with placebo/no treatment. Four of the studies (n = 198) found that use of cervical PGE2 was superior to placebo in decreasing the number of women who did not achieve vaginal delivery within 24 hours (44/100 versus 71/98; RR 0.61, 95% CI 0.47 to 0.79; NNT = 4). In 27 trials including 3734 women, there was a non-significant trend toward decreased risk of caesarean section for women receiving cervical PGE2 (344/1941 versus 360/1793; RR 0.88; 95% CI 0.77 to 1.01). There were no significant increases in risk of hyperstimulation with FHR changes. However, 11 trials with 2531 women demonstrated significant increases in hyperstimulation without FHR changes (67/1344 versus 37/1187; RR 1.59, 95% CI 1.09 to 2.33; NNH = 55). Serious maternal and neonatal morbidity and mortality were infrequently reported and available data revealed similar findings in the PGE2 and placebo groups [15].

The authors identified 29 trials including 3881 women that compared cervical PGE2 with vaginal PGE2. Cervical PGE2 was less effective than vaginal PGE2 in achieving vaginal delivery within 24 hours (11 trials, 2200 women, 410/1122 versus 315/1078; RR 1.26; 95% CI 1.12 to1.41; NNH = 14). There was no difference in any other outcome of interest [15].

#### Subgroup with unfavorable cervix

Compared with placebo, there was a non-significant trend toward fewer caesarean sections among women receiving cervical PGE2 (27 studies, 3716 women, 343/1931 versus 359/1785, RR 0.88; 95% CI, 0.77 to 1.01). In 26 trials with 3586 women whose cervix were unfavorable for induction, there was no difference in caesarean deliveries between women receiving intracervical and intravaginal PGE2 [15].

*Summary: Intracervical PGE2 appears more effective than placebo in achieving vaginal delivery within 24 hours*.

### Oxytocin

Our search identified one Cochrane systematic review which included 61 trials with 12,819 women and evaluated oxytocin for induction of labour [16]. Comparisons were made between intravenous (IV) oxytocin versus placebo/expectant management (25 trials, 6660 women), IV oxytocin versus vaginal prostaglandin (PGE2) (27 trials, 4564 women), IV oxytocin versus intracervical prostaglandins (PGE2) (14 trials, 1331 women), and IV oxytocin versus vaginal PGF2α (3 trials, 291 women) [16].

Three trials including 399 women reported that IV oxytocin, when compared with expectant management, reduced failure to achieve vaginal delivery within 24 hours (16/191 versus 112/208; RR 0.16, 95% CI 0.10 to 0.25; NNT = 3). Meta-analysis of 24 trials including 6620 women found a small but statistically significant increased rate of caesarean delivery for women in the oxytocin group (339/3267 versus 301/3353; RR 1.17, 95% CI 1.01 to1.35; NNH = 66). There was no significant difference in uterine hyperstimulation with or without FHR changes. Use of oxytocin significantly reduced chorioamnionitis (14 studies, 5514 women 144/2720 versus 213/2795; RR 0.69, 95% CI 0.57 to 0.85; NNT = 40); however there was significant heterogeneity among the included trials for this comparison, (I^2 ^= 65%, *P *= 0.001) and the authors' analysis of the studies included in this comparison using the random effects method was not statistically significant. Likewise, NICU admissions were reduced by oxytocin compared to placebo or expectant management, (7 studies, 4387 women, 264/2196 versus 333/2191; RR 0.79, 95% CI 0.68 to 0.92, NNT = 32). However, there was significant between study heterogeneity for this comparison (I^2 ^= 70%, P = 0.0003) and this result was no longer statistically significant when the random effects method was used for analysis. The majority of the studies included in these comparisons required ruptured membranes for entry, likely influencing this result. Data were insufficient to establish conclusions regarding neonatal and maternal mortality or serious morbidity [16].

Three trials including 260 women reported that oxytocin was associated with more failures to achieve vaginal delivery within 24 hours than vaginal PGE2 (73/132 versus 40/128; RR1.77, 95% CI 1.31 to 2.38; NNH = 5). When comparing oxytocin with vaginal PGE2, there was no significant difference in the rates of caesarean section (26 trials, 4514 women, 274/2259 versus 246/2255; RR 1.11, 95% CI 0.94 to 1.30). The incidence of uterine hyperstimulation with fetal heart rate (FHR) changes was very low and not different between groups. Fewer women receiving oxytocin developed chorioamnionitis than those receiving vaginal PGE2 (4 trials, 2742 women, 54/1381 versus 81/1361; RR 0.66, 95% CI 0.47 to 0.92, NNT = 50). Data were insufficient to draw conclusions regarding neonatal and maternal mortality or morbidity; based on limited data, there were no differences between groups [16].

Two studies that included 258 women comparing oxytocin with intracervical PGE2 found that oxytocin was associated with more failure to achieve vaginal deliveries within 24 hours (63/125 versus 46/133; RR 1.47, 95% CI 1.10 to 1.96; NNH = 7). Oxytocin was associated with more caesarean deliveries than intracervical PGE2 (14 studies, 1331 women, 123/643 versus 94/688; RR 1.37, 95% CI 1.08 to 1.74; NNH = 20). There was no significant difference in uterine hyperstimulation with FHR changes. There were not enough data to develop conclusions regarding neonatal and maternal mortality/morbidity [16].

There were only three trials with 291 women that compared oxytocin with PGF2α. None reported on the number of women failing to deliver vaginally within 24 hours. There were no significant differences in uterine hyperstimulation with FHR changes (one trial 23 women) or rates of caesarean delivery (3 trials 280 women). There were no cases of serious neonatal morbidity or perinatal deaths in the two studies that reported this outcome [16].

#### Unfavorable cervix subgroup

Compared with placebo or expectant management, there was no difference in caesarean deliveries among participants with unfavorable cervices (13 trials, 1366 women). Similarly, there was no difference in caesarean deliveries among 1041 women in 15 trials with unfavorable cervices who received oxytocin or vaginal PGE2. However, oxytocin use was more likely to result in caesarean delivery than intracervical PGE2 (10 trials, 1003 women, 107/477 versus 79/526, RR 1.44, 95% CI, 1.12 to 1.86, NNH = 16) [16].

#### Randomised controlled trials published after the search date of systematic reviews

Our search identified one study including 240 women that compared oxytocin with vaginal prostaglandin E2 for premature rupture of membranes at term [17]. In this study, oxytocin was associated with a significantly shorter time from induction to delivery (3.4 +/- 1.5 versus 9.6 +/- 4.7 hours; *p *= 0.02). There was no difference in the risk of caesarean section [17].

*Summary: Oxytocin is more effective than expectant management or placebo but less effective than vaginal and cervical PGE2 in bringing about vaginal delivery within 24 hours. Oxytocin resulted in more caesarean deliveries than cervical PGE2*.

### Amniotomy

We identified one Cochrane systematic review of amniotomy for induction of labour [18]. This review included two studies with 310 total participants. One included study compared women receiving amniotomy with those receiving either oxytocin alone or no intervention. This study was underpowered to detect differences in any outcome of interest and the review concluded that no meaningful results could be drawn from these comparisons. The second included study compared amniotomy alone to a single dose of vaginal prostaglandins for women with a favourable cervix and found a significant increase in the need for oxytocin augmentation in the amniotomy alone group compared with the women receiving PGE2 (260 women, 57/130 versus 20/130; RR 2.85, 95% CI 1.82 to4.46; NNH = 3). There was no difference in caesarean deliveries [18].

#### Subgroup with unfavorable cervix

There were no studies that included participants with unfavorable cervices [18].

*Summary: Compared with vaginal PGE2, amniotomy increases the need for oxytocin augmentation*.

### Oxytocin with amniotomy

Our search identified one Cochrane systematic review including 17 trials with 2566 women comparing IV oxytocin plus amniotomy with other methods for induction of labour [19]. This review compared amniotomy plus oxytocin (in varying doses), with placebo, vaginal prostaglandin E2 or F2α, or amniotomy alone. Oxytocin plus amniotomy resulted in fewer cases of meconium stained amniotic fluid than placebo or no treatment (one trial, 184 participants, 3/92 versus 13/92; RR 0.23, 95% CI 0.07 to 0.78; NNT = 9). There were no other significant differences in our outcomes of interest for this comparison [19].

When compared with vaginal prostaglandins, amniotomy plus IV oxytocin was associated with more postpartum hemorrhage (2 studies, 160 women, 11/80 versus 2/80; RR 5.5, CI 1.26 to 24.07; NNH = 9). One RCT of 100 subjects found that more women were dissatisfied with amniotomy and IV oxytocin than vaginal prostaglandins, (26/50 versus 0/50; RR 53, CI 3.32 to 846.51; NNH = 1). There were no other significant differences between oxytocin plus amniotomy and vaginal prostaglandins [19].

One study with 30 participants compared oxytocin plus amniotomy with cervical prostaglandins. This study was too small to detect any differences in outcomes of interest. Likewise, only two studies with 309 total participants compared oxytocin plus amniotomy with oxytocin alone. These studies were also underpowered to detect differences in any outcome of interest [19].

When compared with those who received amniotomy alone, fewer women who received amniotomy plus IV oxytocin were not delivered vaginally at 24 hours (2 studies, 296 participants, 3/148 versus 24/148; RR 0.13, 95% CI 0.04 to 0.41; NNT = 8). Amniotomy plus IV oxytocin also resulted in significantly fewer instrumental vaginal deliveries than amniotomy alone (2 studies, 510 participants, 57/255 versus 88/255; RR 0.65, CI 0.49 to 0.85; NNT = 995% CI 6 to 20) [19].

#### Unfavorable cervix subgroup

The review included two trials with 106 women who had cervices unfavorable for induction of labor. There was no difference in caesarean birth among women allocated to amniotomy and oxytocin versus vaginal prostaglandins. There were no other trials including women with cervices unfavorable for induction [19].

*Summary: Oxytocin plus amniotomy is more effective than amniotomy alone in achieving vaginal delivery within 24 hours. Oxytocin plus amniotomy may be associated with more postpartum hemorrhage and less maternal satisfaction than vaginal prostaglandins*.

### Vaginal Misoprostol

The Cochrane review of vaginal misoprostol for labour induction included 121 trials [20]. There were no significant difference in vaginal deliveries not achieved with 24 hours among five trials with 769 women that compared vaginal misoprostol with placebo/no treatment. Likewise, in five trials with 777 women, there were no significant differences in hyperstimulation with FHR changes. Compared with placebo/no treatment, vaginal misoprostol was associated with more hyperstimulation without FHR changes (31/313 versus 10/481, 6 trials, 794 women, RR 3.52, 95% CI 1.78 to 6.99, NNH = 19) but with less meconium stained amniotic fluid (6 trials, 814 participants, 27/326 versus 83/488, RR 0.56, 95% CI 0.35 to 0.87, NNT = 14) Vaginal misoprostol reduced the number of participants with a cervix unfavorable or unchanged after 12 to 24 hours (2 studies 107 women, 4/56 versus 41/51, RR 0.10, 95% CI 0.01 to 0.64, NNT = 2). There was no difference in caesarean deliveries in 10 trials that included 1141 women.

Twenty-two trials with 5,229 participants compared vaginal misoprostol with other vaginal prostaglandins for the outcome of vaginal deliveries within 24 hours. Women receiving misoprostol were less likely to not be delivered within 24 hours (22 trials 5229 participants, 920/2550 versus 1179/2679, RR 0.77, 95% CI 0.66 to 0.89, NNT = 10) and were less likely to require oxytocin augmentation (38 trials, 7022 participants, 1355/3465 versus 1794/3557, RR 0.68, 95% CI 0.61 to 0.76, NNT = 7). Meconium-stained amniotic fluid was more common among subjects receiving misoprostol (18 trials, 3991 women, 246/1909 versus 190/2082, RR 1.35, 95% CI 1.13 to 1.61, NNH = 32). Misoprostol increased uterine hyperstimulation without FHR changes (26 trials 4804 women 381/2311 versus 199/2493, RR 1.99, 95% CI 1.41 to 2.79, NNH = 13), although hyperstimulation with FHR changes did not differ (31 trials 5830 women). Vaginal misoprostol reduced the need for oxytocin augmentation (38 trials, 7022 women, 1355/3465 versus 1794/3557, RR 0.68, 95% CI 0.61 to 0.76, NNT = 7) and epidural anesthesia (8 trials, 2141 women, 469/1063 versus 516/1078, RR 0.92 95% CI 0.85 to 0.99, NNH = 27). Caesarean section rates were not significantly different [20].

Compared with cervical PGE2, vaginal misoprostol reduced failure to achieve vaginal delivery within 24 hours (13 trials, 1627 women, 253/814 versus 402/813, RR 0.63, 95% CI 0.56 to 0.71, NNT = 6). Oxytocin augmentation was required less often with misoprostol based on 20 trials including 2316 women, (411/1177 versus 727/1139, RR 0.55, 95% CI, 0.48 to 0.64 NNT = 4) and women receiving misoprostol were less likely to have a cervix unfavorable for induction after 12-24 hours (1 trial, 155 women, 38/76 versus 58/79, RR 0.68, 95% CI 0.52 to0.88, NNT = 5). Women receiving misoprostol were less likely to require epidural anesthesia (2 trials, 321 women, 48/160 versus 75/161, RR 0.64, 95% CI 0.48 to 0.86, NNT = 6). Misoprostol resulted in more uterine hyperstimulation with FHR changes (20 trials, 2224 women, 98/1129 versus 39/1095, RR 2.32, 95% CI 1.64 to3.28, NNH = 22) and without FHR changes (17 trials 2178 women, 194/1097 versus 96/1081, RR 1.95, 95% CI 1.57 to 2.42, NNH = 12). Increased rates of meconium-stained amniotic fluid were observed with use of misoprostol (14 trials 2018 women, 161/1015 versus 123/1003, RR 1.29, 95% CI 1.04 to1.59, NNH = 29). There were no other statistically significant differences in perinatal or maternal outcomes [20].

Compared with oxytocin, vaginal misoprostol reduced the likelihood of participants not being delivered vaginally within 24 hours (10 trials, 1397 women, 135/690 versus 226/707, RR 0.65 95% CI.47 to 0.90, NNT = 9). Vaginal misoprostol was associated with increased uterine hyperstimulation with FHR changes (9 trials with 1419 women 49/690 versus 28/729, RR 1.87, 95% CI 1.20 to 2.91, NNH = 31) and without FHR changes (15 trials 2050 women, 218/1009 versus 102/1041, RR 2.24, 95% CI 1.82 to 2.77, NNH = 9). Women receiving misoprostol were more likely to experience gastrointestinal side effects than those receiving oxytocin (4 trials 334 women, 15/170 versus 2/164, RR 5.04, 95% CI 1.51 to 16.86, NNH = 21) Caesarean deliveries were less likely among women receiving vaginal misoprostol (25 trials 3074 women, 258/1527 versus 364/1547, RR 0.76 95% CI 0.60 to 0.96, NNT = 18) as were instrumental vaginal deliveries (13 trials, 1639 women, 69/810 versus 96/829, RR 0.74 95% CI 0.56 to 0.99, NNT = 34). Infants born to women receiving misoprostol were less likely to have Apgar score < 7 at 5 minutes of life (13 trials, 1906 women, 22/938 versus 41/968, RR 0.56, 95% CI 0.34 to 0.92, NNT = 54). There were no differences in other maternal or fetal outcomes of interest [20].

#### Unfavorable cervix subgroup

Compared with placebo, there was no difference in caesarean deliveries for women receiving vaginal misoprostol (7 trials, 862 women). There was no difference in the likelihood of caesarean deliveries among women receiving misoprostol or women receiving vaginal PGE2 (28 trials, 5832 women), cervical PGE2 (21 trials, 2499 women) or oxytocin (14 trials, 1598 women) [20].

*Summary: Vaginal misoprostol is more likely to result in vaginal delivery within 24 hours than vaginal or cervical PGE2 or oxytocin but is associated with increased uterine hyperstimulation. Compared with IV oxytocin, vaginal misoprostol may reduce the likelihood of caesarean delivery*.

### Oral Misoprostol

The Cochrane review of oral misoprostol compared to other methods of labour induction included 56 RCTs with a total of 11,590 participants [21]. There were seven trials with 669 women that compared oral misoprostol to placebo. Women assigned to receive oral misoprostol were more likely to give birth vaginally within 24 hours (1 trial, 96 women, 3/47 versus 20/49; RR 0.16, CI 0.05 to 0.49; NNT = 3). Oral misoprostol was also associated with lower caesarean section rates than placebo (six trials, 629 women, 31/312 versus 51/317; RR 0.61, 95% CI 0.41 to 0.93; NNT = 16). Fewer women receiving oral misoprostol required oxytocin augmentation (6 trials, 535 subjects, 63/266 versus 181/269; RR 0.35, 95% CI 0.28 to 0.44; NNT = 3) [21].

Ten trials with a total of 3368 women compared oral misoprostol with vaginal PGE2. Fewer women assigned to receive oral misoprostol required caesarean delivery (10 trials, 3368 participants, 340/1599 versus 467/1769; RR 0.87, 95% CI 0.77 to 0.98; NNT = 30). In two trials including 930 subjects, more women assigned to oral misoprostol had an unfavourable cervix after 24 hours (74/470 versus 51/460; RR 1.41, 95% CI 1.01 to 1.96; NNH = 22). There were no statistically significant differences between the groups in any of the other outcomes, including hyperstimulation with and without FHR changes and the frequency of meconium-stained amniotic fluid. There was significant heterogeneity (*P *= 0.002) among studies comparing oral misoprostol and vaginal PGE2 for the outcome of uterine hyperstimulation without FHR changes, likely related to the differing doses of oral misoprostol used in the included studies [21].

Four trials with 681 women compared oral misoprostol to intracervical PGE2. Fewer women assigned to oral misoprostol group failed to deliver vaginally within 24 hours, although this finding was of borderline statistical significance (2 trials, 391 participants, 81/196 versus 100/195; RR 0.81, 95% CI 0.65--1.00; NNT = 11). Oral misoprostol was associated with more uterine hyperstimulation with FHR changes (3 trials, 490 women, 12/245 versus 3/245; RR 3.57, 95% CI 1.11 to 11.54; NNH = 32). There was a trend of borderline significance toward more hyperstimulation without FHR changes with oral misoprostol (1 trial, 190 women, 8/95 versus 0/95; RR 17.01, 95% CI 1.00 to 290.42). There were no differences in any other outcome of interest [21].

Eight trials including 1026 women compared oral misoprostol with IV oxytocin. Meconium staining of the amniotic fluid was seen more frequently in the misoprostol group (6 trials, 916 women, 47/477 versus 24/439; RR 1.72, 95% CI 1.08 to 2.74; NNH = 26), but oral misoprostol was not associated with any other differences in adverse fetal, neonatal or maternal outcomes [21].

Twenty-six trials with 5096 participants compared oral with vaginal misoprostol. The oral route of administration was associated with more frequent use of oxytocin (22 trials, 4557 women, 1301/2279 versus 1151/2278; RR 1.19, 95% CI 1.06 to 1.34; NNH = 11), but there was no difference in vaginal delivery within 24 hours. There were significantly lower rates of uterine hyperstimulation without FHR changes with oral regimens (9 trials, 1420 women, 85/698 versus 146/722; RR 0.58, 95% CI 0.35 to 0.96; NNT = 12), but the rate of hyperstimulation with FHR changes was not different between the two groups. Fewer babies born to mothers who received oral misoprostol had Apgar scores less than 7 at five minutes of life (14 trials, 3270 women 37/1638 versus 57/1632; RR 0.65, 95% CI 0.44 to 0.97; NNT = 82). There was no difference in caesarean deliveries in 25 trials with 5096 women [21].

#### Unfavorable cervix subgroup

There were no studies comparing oral misoprostol with placebo, vaginal PGE2, intracervical PGE2, or oxytocin that reported on caesarean deliveries among women with unfavorable cervices. Among women with unfavorable cervices who were randomized to receive oral misoprostol or vaginal misoprostol there were no differences in caesarean deliveries among primiparous (2 studies, 85 participants) or multiparous (1 study, 24 participants) subjects [21].

#### Randomised controlled trials published after the search date of systematic reviews

Subsequent to the search date of the Cochrane review, we identified one study with 87 participants that compared oral misoprostol with placebo [22]. This study found oral misoprostol superior to placebo in achieving delivery within 24 hours (19/43 versus 6/44, *P *= 0.024, NNT = 4). An additional study that compared oral misoprostol with PGE2 found that more women receiving misoprostol delivered vaginally within 12 hours, although vaginal deliveries within 24 hours did not differ [23]. More women receiving oral misoprostol were satisfied with their induction method [23].

*Summary: Oral misoprostol reduced caesarean sections compared with vaginal PGE2 and placebo. Compared with vaginal misoprostol, oral misoprostol is associated with fewer contractile abnormalities, but more need for oxytocin augmentation*.

### Buccal or sublingual misoprostol

Our search uncovered three systematic reviews comparing sublingual or buccal misoprostol with other methods of labour induction [24-26]. The Cochrane review by Muzonzini[24] and colleagues and the review by Bartusevicius and colleagues[25] both included the same three studies with a total of 507 women and reached similar conclusions. Two of the studies with a total of 350 women compared buccal or sublingual misoprostol (50 μg) to oral misoprostol (50 or 100 μg) and one study with 157 participants compared buccal and vaginal misoprostol. Neither review found significant differences in any outcome of interest [24,25].

#### Unfavorable cervix subgroup

The Cochrane reviewers did not conduct any subgroup analyses according to cervical status [24].

#### Other systematic reviews

In 2008 Souza and colleagues published a systematic review of studies comparing sublingual or buccal misoprostol with vaginal misoprostol for induction of labour. This review included five studies with 740 subjects [26]. The authors found no significant differences in the rates of vaginal delivery not achieved within 24 hours, hyperstimulation, or cesarean deliveries. There were more cases of uterine tachysystole, defined as more than five contractions in 10 minutes for at least 20 minutes, among women assigned to sublingual misoprostol (5 trials, 740 women, 42/368 versus 26/372; OR 1.70, 95% CI 1.02 to 2.83; NNH = 24, 95% CI 10 to 771), although there was significant heterogeneity among studies included in this comparison (*P *= 0.04) [26].

#### Randomised controlled trials published after the search date of systematic reviews

Our search identified two additional studies carried out subsequent to the search dates of these systematic reviews. One of these studies compared sublingual misoprostol 50 μg with amniotomy and oxytocin for induction of labour among 50 women at term with favourable cervices [27]. This study was terminated early when an interim analysis revealed that significantly fewer women allocated to sublingual misoprostol delivered within 24 hours (15/22 versus21/21; RR 0.68, 95% CI 0.51 to 0.91; NNH = 3). There were no differences in other maternal or fetal outcomes, although maternal satisfaction was significantly higher with sublingual misoprostol. The second study included 150 women and compared 50 μg misoprosol by oral, vaginal, or sublingual routes [28]. This study found that the induction to delivery interval was significantly decreased among women receiving sublingual misoprostol compared with the vaginal and oral routes (13.3 hours versus 16.1 hours [oral] versus 15.1 hours [vaginal]). Fewer babies born to mothers receiving sublingual misoprostol had Apgar scores less than seven at one minute (0/50 versus 6/100, *P *= 0.003, NNT = 17) [28].

*Summary: Compared with vaginal misoprostol, administration of misoprostol by the buccal or sublingual route increases uterine tachysystole*.

### Mechanical methods

Our search identified three systematic reviews evaluating mechanical methods for induction of labour. The Cochrane review studied mechanical methods including laminaria tents, synthetic equivalents such as Dilapan, Foley catheters, and other types of balloon catheter for induction of labour. It included 45 RCTs that compared mechanical methods with PGE2, misoprostol, oxytocin, and placebo. Most trials had small sample sizes [29].

The authors did not find any advantage of mechanical methods compared with placebo or no treatment in the pre-specified outcomes of vaginal delivery not achieved within 24 hours or caesarean deliveries (1 trial, 48 women). There was no difference in caesarean deliveries between women receiving mechanical methods and women receiving placebo or no treatment (6 studies, 416 participants). There was no difference in any other outcome of interest, including chorioamnionitis (1 trial, 240 participants) and endometritis (2 trials, 288 participants) [29].

More subjects allocated to mechanical methods failed to deliver vaginally within 24 hours than those assigned to vaginal PGE2 (1 trial, 109 participants, 43/59 versus 21/50, RR 1.74, CI 1.21 to 2.49; NNH = 3), and more women allocated to mechanical methods required oxytocin augmentation (2 trials, 169 women, 30/89 versus 9/80, RR 2.90, 95% CI 1.40 to 6.00, NNH = 5), although this finding should be interpreted with caution, as there was significant heterogeneity between the 2 studies included in this comparison (*P *= 0.0008). Mechanical methods were less likely to result in uterine hyperstimulation with FHR changes in 6 trials with 484 women, (0/246 versus 14/238, RR 0.14, 95% CI 0.04 to 0.53, NNT = 20) and without FHR changes in 8 trials with 580 women (6/293 vs 28/287, RR 0.26, 95% CI 0.13 to 0.54, NNT = 14). There was no difference in caesarean deliveries between mechanical methods and vaginal PGE2 (12 trials, 786 women), but mechanical methods were associated with reduced need for instrumental vaginal deliveries (5 trials, 378 women, 36/192 versus 53/186, RR 0.65, 95% CI 0.46 to 0.93, NNT = 11) [29].

More women assigned to mechanical methods did not achieve vaginal delivery within 24 hours than those assigned to cervical PGE2 (1 trial, 100 participants, 34/50 versus 20/50; RR 1.70, CI 1.15 to 2.50; NNH = 3), and more women allocated to mechanical methods required oxytocin augmentation (1 trial, 185 women, 84/90 versus 63/95, RR 1.41, 95% CI 1.21 to 1.64, NNH = 3). However, there was no difference in caesarean sections in 12 trials that included 1614 women. Compared with cervical PGE2, mechanical methods were associated with less endometritis (4 trials, 693 participants, 9/352 versus 34/341 RR 0.26, 95% CI 0.13 to 0.52, NNT = 14). However, in 3 studies with 619 women that compared mechanical methods to cervical PGE2, there were more neonatal infections in babies born to mothers who had received mechanical methods compared to cervical PGE2 (24/316 versus 9/303, RR 2.45 95% CI 1.18, to 5.07, NNH = 24) [29].

Analysis of four studies with 198 women comparing mechanical methods with oxytocin found that mechanical methods resulted in fewer caesarean deliveries (18/103 versus 30/95; RR 0.55, 95% CI 0.33 to 0.91; NNT = 8). There was no difference in hyperstimulation without FHR changes, postpartum hemorrhage, or serious maternal morbidity or death in 1 trial with 60 women. No other outcomes could be evaluated for this comparison [29].

Four studies including 618 women compared mechanical methods to vaginal misoprostol. There were no statistical differences in the likelihood of achieving vaginal delivery within 24 hours (2 studies, 234 women) or in caesarean deliveries (4 studies, 618 women). There was reduced risk for uterine hyperstimulation with FHR changes seen in three trials including 434 women comparing mechanical methods to vaginal misoprostol (8/226 versus 19/208; RR 0.41, CI 0.20 to 0.87; NNT = 19). There were no differences noted in infectious morbidity or neonatal outcomes [29].

#### Unfavorable cervix subgroup

There was no difference in caesarean deliveries among 396 women enrolled in five randomized controlled trials comparing mechanical methods with placebo or no treatment, in 10 trials with 738 women comparing mechanical methods with vaginal PGE2 or in 12 trials with 1614 women comparing mechanical methods with intracervical PGE2. There was no difference in cesarean sections in three trials with 482 participants comparing mechanical methods with vaginal misoprostol. In the subgroup of women with unfavorable cervices, mechanical methods were less likely to result in caesarean delivery than oxytocin (3 trials, 178 women, 15/93 versus 27/85, RR 0.50, 95% CI, 0.29 to 0.87, NNT = 7) [29].

#### Other systematic reviews

Our search identified a second systematic review that compared mechanical methods of labour induction with PGE2, misoprostol, hyaluronidase or placebo [30]. This systematic review, which included 30 randomised, controlled trials with a total of 4468 participants, focused on the outcomes of maternal and neonatal infectious morbidity. The authors defined maternal infectious morbidity as maternal temperature greater than 38°C, endometritis or chorioamnionitis. They defined neonatal infectious morbidity as fever, suspected or proven sepsis, or need for antibiotics. Controls were the pooled group of women who had received other pharmacologic methods of labour induction. Compared with controls, women undergoing labour induction with mechanical methods were more likely to experience infectious morbidity (30 studies, 4468 participants, 252/2220 versus 188/2248; OR 1.38, 95% CI 1.12 to 1.68; NNH = 36). The authors reported no significant heterogeneity for this comparison. Similarly, infants born to mothers undergoing mechanical methods of induction were more likely to experience neonatal infectious morbidity than infants born to mothers undergoing induction with pharmacologic methods (8 trials, 1775 women, 40/893 versus 18/882; OR 2.03, 95% CI 1.19 to 3.51; NNH = 50). The authors reported that there was no significant heterogeneity for this comparison [30].

A third systematic review compared mechanical methods (Foley catheter balloon) with locally applied prostaglandins (vaginal PGE2, cervical PGE2 and vaginal misoprostol) [31]. This systematic review included 27 randomized controlled trials that included 3532 participants. When compared with all locally applied prostaglandins (LAPG) combined, there were no differences between mechanical methods and prostaglandins in caesarean deliveries (27 trial, 3532 participants), participants with cervices that were unfavorable or unchanged after 12 to 24 hours (6 trials, 613 participants), ripening to delivery interval (13 trials, 1270 participants), vaginal deliveries within 12 to 24 hours (13 trials, 1779 women), maternal fevers (19 trials, 2421 women), 5-minute Apgar scores less than 7 (14 trials, 1661 women), meconium staining (13 trials 1841 women), or admission of the neonate to a NICU (12 trials, 1796 women). Women who received LAPG were less likely to require oxytocin augmentation than those receiving mechanical methods (16 trials, 1644 participants, RR 0.73, 95% CI 0.62 to 0.86, P = 0.0002), but were more likely to experience excessive uterine activity, defined as tachysystole, hypertonus, or hyperstimulation syndrome (21 trials, 2661 participants, 244/1306 versus 147/1355, RR, 2.35; 95% CI, 1.41 to 3.90; *P = *.001, NNH for locally applied prostaglandins when compared with mechanical methods = 7). There was significant heterogeneity noted for the outcomes of excessive uterine activity, vaginal delivery within 12-24 hours, and for need for oxytocin augmentation [31].

The authors conducted subgroup analyses comparing mechanical methods with vaginal PGE2, cervical PGE2, and vaginal misoprostol. They found that mechanical methods were associated with a longer ripening to delivery interval than cervical PGE2 (5 trials, 552 subjects, weighted mean difference [WMD] 5.48 hours, 95% CI 2.79 to 8.16, *P *< 0.0001) and vaginal PGE2 (2 trials, 118 subjects, WMD,4.55 hours, 95% CI, 0.33 to 8.77; *P = *.03). Cervical PGE2 was associated with a higher risk for caesarean deliveries than mechanical methods in seven trials with 896 women (OR 1.27, 95% CI 1.01 to 1.59, *P *= 0.04). Vaginal misoprostol was associated with increased risk for excessive uterine activity compared with Foley balloon in 13 trials with 1847 participants, RR 3.41, 95% CI, 1.97 to 5.90; *P *= 0.0001) [31].

#### Randomised controlled trials published after the search date of systematic reviews

We included one additional trial that was published subsequent to the search date of the third systematic review [32]. This trial randomly assigned 240 women to receive vaginal misoprostol 25 μg or a Foley catheter for labor induction. This study found that the Foley catheter was associated with a longer induction to vaginal delivery interval than the vaginal misoprostol (20.2 hours versus 17.3 hours, *P *= 0.016). There were no significant differences in other outcomes of interest [32].

*Summary: Mechanical methods are less likely to result in uterine hyperstimulation than PGE2 or vaginal misoprostol, but may be associated with increased maternal and neonatal infectious morbidity*.

### Membrane Sweeping

Our search identified one Cochrane systematic review of 22 trials which included 2797 subjects that compared membrane sweeping with oxytocin, PGE2, or no treatment [33]. There was no difference in rates of caesarean deliveries, serious neonatal morbidity, perinatal death, serious maternal or neonatal infections when comparing membrane sweeping with no treatment. A policy of routine membrane sweeping from 37 weeks onward reduced the likelihood of gestation continuing to both 41 (6 studies, 937 women, 77/473 versus 129/464; RR 0.59, 95% CI 0.46 to 0.74; NNT = 9) and 42 (6 studies, 722 women, 12/365 versus 43/357; RR 0.28, 95% CI 0.15 to 0.50; NNT = 12) weeks' gestation. Membrane sweeping was associated with reduced likelihood of not being in labour within 48 hours (five studies, 726 women, 234/367 versus 298/359; RR 0.77, 95% CI 0.70 to 0.84; NNT = 6). Membrane sweeping was also associated with reduced risk for not being delivered within one week (9 studies, 1375 women, 320/695 versus 440/680; RR 0.71, 95% CI 0.65 to 0.78; NNT = 6). Membrane sweeping was associated with more vaginal bleeding (3 trials, 391 women, 35/200 versus 18/191; RR 1.75, 95% CI 1.08 to 2.83; NNH = 15) and more maternal discomfort (2 studies, 320 women, 94/163 versus 32/157; RR 2.83, 95% CI 2.03 to 3.96; NNH = 3) compared with no treatment. Data comparing membrane sweeping with PGE2 and with oxytocin were insufficient to draw conclusions of relative efficacy [33].

#### Unfavorable cervix subgroup

There was no difference in caesarean sections among women allocated to membrane sweeping versus no treatment (3 trials, 200 women) nor in two trials with 252 women comparing membrane sweeping with vaginal prostaglandins nor in one trial with 69 women comparing membrane sweeping with oxytocin [33].

#### Randomised controlled trials published after the search date of systematic reviews or awaiting classification

Among the studies awaiting classification in the Cochrane review were five high quality RCTs [34-38]. In addition, we identified one additional high quality RCT that was published after the Cochrane review's search date [39]. Of these, five studies with 1700 participants compared membrane sweeping with no treatment or vaginal exam alone [35-39]. In a meta-analysis that added our independently-extracted data from the two studies [36,37] that evaluating the effect of membrane sweeping on post-term gestations to the data reported in the Cochrane review, membrane sweeping significantly decreased the number of pregnancies progressing to 42 weeks' gestation (8 studies, 1874 participants, 102/902 versus 194/862, RR 0.53, 95% CI 0.43 to 0.65, NNT = 10). There was no significant heterogeneity for this comparison. Thus the addition of these two new trials did not alter the conclusion reached by the Cochrane review.

DeMiranda found that membrane sweeping significantly reduced the time from randomization to delivery by one day (3.50 versus 4.47 days, mean difference 0.97 days; 95% CI 0.60 to 1.35) [36]. In a more recent study involving 351 women, Yildirim and colleagues found that membrane sweeping significantly increased the likelihood of spontaneous labor by 41 weeks' gestation (162/179 versus 118/167, P = 0.0001) [38]. By contrast, Hamdan found that membrane sweeping did not increase the proportion of women planning trial of labor after prior cesarean section (TOLAC) who entered spontaneous labor [39]. The Hill study was designed to test whether membrane sweeping increases prelabour rupture of membranes, but found no overall difference in this outcome [37]. One study with 60 participants compared membrane sweeping with a single dose of intracervical PGE2 [34]. Use of cervical PGE2 resulted in a significantly shorter intervention to delivery interval than did membrane sweeping (26.23 hours versus 19.15 hours, P < 0.01) [34].

*Summary: Membrane sweeping reduces the risk of post-term gestation*.

## Complementary and Alternative Medicine Methods

### Castor Oil

Our search identified one systematic review of the efficacy of castor oil for induction of labour [40]. The authors of this Cochrane review identified only one study with 100 subjects comparing castor oil with no treatment. The trial was judged to be of poor methodologic quality due to methods of allocation. There were no observed differences in rates of caesarean delivery, meconium stained fluid, or Apgar less than 7 at five minutes. More women receiving castor oil reported experiencing nausea (52/52 versus 0/48; RR 97.08, 95% CI 6.16 to 150.34; NNH = 1) [40].

#### Unfavorable cervix subgroup

In one trial with 100 women, there was no difference in caesarean deliveries between women who received castor oil or no treatment [40].

*Summary: Compared with no treatment, castor oil is associated with increased maternal side effects*.

### Acupuncture

Our search identified a Cochrane systematic review that included 3 trials with 212 women that focused on acupuncture for induction of labour [41]. Compared with standard care (oxytocin, prostaglandins, or "routine care"), more women undergoing acupuncture did not require the use of other induction methods (2 trials, 147 women, 49/73 versus 34/74; RR 1.45, 95% CI 1.08 to1.95; NNT = 5). No differences were found in time to delivery, rates of caesarean delivery, instrumental vaginal delivery, or epidural anesthesia. Fetal or neonatal outcomes were not estimable [41].

#### Unfavorable cervix subgroup

There was no data in the included trials concerning the effect of acupuncture for labor induction in women with unfavorable cervices [41].

#### Randomised controlled trials published after the search date of systematic reviews

We identified three further trials with 684 participants, three published after the search date of the systematic review [42-44] and one that was not identified by the Cochrane search [45]. These studies did not reveal any differences between acupuncture and placebo or no treatment in any outcome of interest.

*Summary: The use of acupuncture for induction of labour is investigational; no advantages for this method have been demonstrated*.

### Breast Stimulation

Our search identified one systematic review that combined six studies with 719 subjects that evaluated breast stimulation for labour induction [46]. There were no differences in cesarean deliveries, meconium staining, or uterine hyperstimulation when comparing breast stimulation with no treatment. Breast stimulation decreased the number of women who were not in labour within 72 hours (4 studies, 437 women, 136/217 versus 206/220; RR 0.67, 95% CI 0.60 to 0.74; NNT = 4). Breast stimulation was associated with less postpartum hemorrhage (2 studies, 300 women, 1/150 versus 9/150; RR 0.16, 95% CI 0.03 to 0.87; NNT = 20). There were more perinatal deaths among pregnancies assigned to breast stimulation than to no treatment, although this difference was not statistically significant (3 studies, 337 participants, 3/167 versus 0/170; RR 8.17, 95% CI 0.45 to 147.8). This result should be interpreted with caution, as all of the deaths occurred in a single trial conducted among high risk women in a developing country [46].

Two studies with a total of 99 subjects compared breast stimulation with oxytocin. There were no differences in caesarean deliveries. In one trial with 37 women, more women assigned to breast stimulation were not in labour within 72 hours compared with those who were allocated to the oxytocin group, although this difference was of borderline statistical significance (10/17 versus 5/20; RR 2.35, 95% CI 1.00 to 5.54). There were no differences in uterine hyperstimulation or meconium staining. There were three perinatal deaths in the breast stimulation group versus one in the oxytocin group, a non-significant difference. All deaths were from the same trial conducted among high risk women in a developing-world setting [46].

#### Unfavorable cervix subgroup

There was no information on cesarean deliveries in the one included trial that included women with unfavorable cervices [46].

*Summary: Breast stimulation may reduce the number of women not in labour within 72 hours compared to no treatment but is less effective than oxytocin for this outcome. More research is needed to evaluate the safety of breast stimulation*.

### Intercourse

The Cochrane review of intercourse for induction of labour included one study with 28 subjects [47]. Participants were assigned to have intercourse nightly for three nights versus no intercourse. There were no differences in delivery within three days or five minute Apgar less than seven [47].

#### Unfavorable cervix subgroup

There was no information on cervical status or cesarean deliveries in the one included study [47].

*Summary: There is not enough evidence to evaluate the efficacy and safety of intercourse for induction of labour*.

### Homeopathic methods

Our search identified one systematic review of two studies with 133 participants that compared homeopathic herbs for labour induction with placebo [48]. Only one of the studies included in the Cochrane review reported on the pre-specified clinical outcomes of interest. That study included 40 subjects and reported no difference in rates of vaginal delivery not achieved with 24 hours, caesarean deliveries, operative vaginal delivery, need for oxytocin augmentation of labour, or length of labour [48].

#### Unfavorable cervix subgroup

There was no information in the included study on cervical status [48].

*Summary: There is not enough evidence to evaluate the risks and benefits of homeopathy for induction of labour*.

### Hypnotic Relaxation

We identified one quasi-randomised study of hypnotic relaxation for induction of labour in post-term pregnancies [49]. Forty women were assigned to hypnotic relaxation and an equal number to no intervention based on alternate days of the week. Controls were also chosen based on the baseline characteristics of parity, gestational age, and cervical status. There were no differences in delivery within 24 hours or time to delivery. The authors did not report any other outcomes [49].

#### Unfavorable cervix subgroup

There was no information on the effect of hypnotic relaxation among women with unfavorable cervices [49].

*Summary: Compared to no intervention, hypnotic relaxation did not affect likelihood of delivery within 24 hours. Data were insufficient to evaluate any other outcome*.

## Investigational Methods

### Extra-amniotic prostaglandins

Extra-amniotic placement of prostaglandins has been studied as a combination of a mechanical method (Foley catheter) with a pharmacologic method (prostaglandins) [50]. The prostaglandin is introduced into the extra-amniotic space via the catheter. Our search identified one systematic review comparing extra-amniotic prostaglandins with other methods for induction of labour. This review included 12 studies which compared extra-amniotic PGE2 or PGF2α with extra-amniotic placebo, vaginal prostaglandins, intracervical prostaglandins, IV oxytocin, vaginal misoprostol, or mechanical methods. Because of the wide variety of comparisons, with fewer than 200 participants in each of the individual comparisons, evaluation of this modality compared to other methods was limited. Three RCTs with 167 women compared extra-amniotic prostaglandins with placebo. Women receiving extra-amniotic prostaglandins were less likely to require oxytocin augmentation (34/84 versus 66/83; RR 0.51, 95% CI 0.39 to 0.67; NNT = 3). Extra-amniotic PGE2 reduced the likelihood of cervix unfavourable for induction after 12 to 24 hours compared with Foley catheter alone (1 trial, 187 participants, 27/90 versus 49/97; RR 0.59, 95% CI 0.41--0.86; NNT = 5) [50].

The authors found that women allocated to extra-amniotic F2α prostaglandins were more likely to be not vaginally delivered within 24 hours than women receiving vaginal misoprostol (1 trial, 152 women, 34/76 versus 14/76, RR 2.43; 95% CI 1.42 to 4.15; NNH = 3). Women were more likely to be satisfied with extra-amniotic prostaglandins compared with vaginal PGE2 (1 trial, 62 women, mean difference 4.40, 95% CI 3.50 to 5.30). Evaluation of other maternal and fetal outcomes was limited due to the small numbers of included women and many different types of comparison [50].

#### Unfavorable cervix subgroup

There was no difference in caesarean deliveries among women receiving extra-amniotic PGE2 versus extra-amniotic placebo (2 trials, 60 participants) nor between women receiving extra-amniotic PGF2α and extra-amniotic placebo (1 trial, 25 participants). There was no difference in caesarean sections between women receiving extra-amniotic PGE2 and vaginal PGE2 (3 trials with 142 women), or between women receiving extra-amniotic PGE2 and intracervical PGE2 (1 trial 194 women). One trial with 30 participants with unfavorable cervices found no difference in caesarean deliveries between women receiving extra-amniotic PGE2 and oxytocin. In one trial with 77 women there was no difference in caesarean sections between women receiving an intracervical Foley catheter and extra-amniotic PGE2. There was no data for comparison of vaginal or oral misoprostol with extra-amniotic PGE2 among women with unfavorable cervices [50].

*Summary: Data are insufficient to recommend extra-amniotic prostaglandins*.

### Intravenous prostaglandins

In the 1970s and 1980s, IV prostaglandins were investigated as a potential option for induction of labour [51]. Our search identified one systematic review comparing IV prostaglandins (PGE_2 _or IV PGF2α) with oxytocin. This Cochrane review included thirteen trials, with a total of 1165 women, which were carried out between 1970 and 1987. Compared with IV oxytocin, the use of IV prostaglandin was associated with higher rates of uterine hyperstimulation both with FHR changes (5 trials, 390 women, 9/199 versus 0/191; RR 6.76, 95% CI 1.23 to 37.11, NNH = not estimable) and without FHR changes (5 trials 318 women, 17/159 versus 4/159; RR 4.25, 95% CI 1.48 to 12.24, NNH = 13). However, there was no difference in caesarean deliveries. Maternal side effects, defined as gastrointestinal symptoms, fever, and thrombophlebitis were more common in the IV prostaglandin group (8 trials, 940 women, 87/474 versus 22/466; RR 3.75, 95% CI 2.46 to 5.70, NNH = 8). There were four perinatal deaths among 491 women who received IV prostaglandins compared to no perinatal deaths among 483 women who received IV oxytocin. This difference was not statistically significant. There were no differences in NICU admissions and Apgar scores. There was no difference in vaginal deliveries within 24 hours [51].

#### Unfavorable cervix subgroup

In 1 trial with 100 primiparous participants, there was no difference in caesarean section among women receiving IV prostaglandins and women receiving IV oxytocin [51].

*Summary: Intravenous prostaglandins have no advantages and increase maternal side effects compared to other methods of induction. This method of induction of labour has not entered into general use and is of historical interest only*.

### Oral prostaglandins (excluding misoprostol)

Our search identified one Cochrane systematic review comparing oral PGE2 with other methods of induction of labour [52]. This review included 19 studies with 2588 women. Included studies compared oral PGE2 with placebo, cervical or vaginal PGE2, or oral or IV oxytocin, with or without amniotomy. There was no difference in the number of participants who achieved vaginal delivery within 24 hours between women receiving oral PGE2 and controls who received IV oxytocin. Oral PGE2 was associated with fewer caesarean deliveries than placebo (3 studies, 195 women, 14/105 versus 20/90; RR 0.54, 95% CI 0.29 to 0.98; NNT = 10). There was no difference in caesarean delivery between subjects induced with oral PGE2 and other methods of induction. Oral PGE2 was associated with increased vomiting compared to IV oxytocin (3 studies, 305 women, 25/150 versus 4/155; RR 5.56, 95% CI 2.15 to14.38; NNH = 9). More women allocated to oral PGE2 experienced diarrhea (2 studies, 236 women, 6/114 versus 0/122; RR 8.13, 95% CI 1.03 to 63.93; NNH = not estimable) compared with IV oxytocin. There were no significant differences in other maternal or fetal outcomes in any of the other comparison groups [52].

#### Unfavorable cervix subgroup

Among women with unfavorable cervices, oral PGE2 was associated with fewer caesarean deliveries than placebo (3 studies, 195 women, 14/105 versus 20/90; RR 0.54, 95% CI 0.29 to 0.98; NNT = 10). There was no difference between caesarean deliveries among women receiving oral prostaglandins and those receiving vaginal prostaglandins (2 trials, 63 women), cervical prostaglandins (1 trial, 50 participants), or oxytocin (3 trials, 171 women) [52].

*Summary: Oral prostaglandins are associated with increased maternal vomiting and diarrhea compared with IV oxytocin*.

### Mifepristone

Our search uncovered one systematic review of mifepristone for induction of labour combining 10 trials including 1108 women [53]. The authors found that mifepristone was superior to placebo in achieving a favourable cervical score or initiating labour within 48 hours (4 studies, 293 women, 75/152 versus 27/171; RR 2.41, 95% CI 1.70 to3.42, NNT = 4). Compared to placebo, mifepristone reduced the risk for caesarean section (9 trials, 1043 women, 163/661 versus 113/382; RR 0.74, 95% CI 0.60 to 0.92; NNT = 14), but increased the risk for instrumental vaginal delivery (7 trials, 814 women, 139/540 versus 47/274; RR 1.43, 95% CI 1.04 to1.96; NNH = 14). Compared to placebo, mifepristone increased the likelihood of FHR abnormalities (5 trials, 721 women, 101/493 versus 35/228; RR 1.60, 95% CI 1.12 to 2.29; NNH = 11), but did not adversely affect neonatal outcomes [53].

The reviewers included one study comparing mifepristone to oxytocin for induction of labor among women with PROM at term. That study found that compared with oxytocin, mifepristone decreased the proportion of women who were delivered vaginally within 24 hours (1 study, 65 participants, 17/33 versus 25/32, RR 0.66, 95% CI 0.45 to 0.96, NNH = 3). Mifepristone was associated with increased FHR tracing abnormalities (9/33 versus 2/32, RR 4.46, 95% CI 1.02 to 18.66, NNH = 4) and neonatal ICU admissions (11/33 versus 3/32, RR 3.56, 95% CI 1.09 to 11.58, NNH = 4) [53].

#### Unfavorable cervix subgroup

Among women with unfavorable cervices, mifepristone reduced the likelihood of caesarean section compared with placebo (8 trials, 919 participants, 153/599 versus 96/320, RR 0.77 95% CI 0.61 to 0.96, NNT = 15) [53].

*Summary: The use of mifepristone for labour induction is currently investigational*.

### Oestrogens

Our search uncovered one systematic review of oestrogens with or without amniotomy for induction of labour that included seven RCTs and a total of 465 women [54]. Five studies including 306 subjects compared oestrogen with placebo. There were no differences in rates of caesarean deliveries, operative vaginal deliveries or uterine hyperstimulation with or without FHR changes between groups. There were no differences between oestrogens and vaginal prostaglandins in caesarean deliveries, uterine hyperstimulation with or without FHR changes, or epidural analgesia (1 trial, 60 women). There were no differences between oestrogens and cervical prostaglandins in cesarean deliveries or instrumental vaginal deliveries (2 trials, 151 women). There was no difference between oestrogens and cervical prostaglandins in serious maternal complications or NICU admissions in 1 trial with 85 women. There were no differences between oestrogens and oxytocin in caesarean deliveries or operative vaginal deliveries in one trial including 66 women [54].

#### Unfavorable cervix subgroup

In three trials including 162 women, there was no difference in caesarean sections between women receiving oestrogens and placebo. There was no difference in caesarean deliveries between women receiving oestrogens and vaginal prostaglandins (1 trial, 60 women), cervical prostaglandins (one trial, 66 women), extra-amniotic prostaglandins (one trial, 30 women), or oxytocin (one trial, 66 women) [54].

*Summary: The use of oestrogens for induction of labour is currently investigational*.

### Corticosteroids

In a Cochrane review, Kavanagh and colleagues identified eight studies examining the use of corticosteroids for labour induction [55]. Seven of these did not meet the authors' inclusion criteria. The one included trial had 66 women and evaluated post-term pregnancies which were randomly assigned to receive two dexamethasone injections (12 and 24 hours prior to oxytocin infusion) or no treatment prior to oxytocin. There were no differences in caesarean deliveries, uterine hyperstimulation with or without FHR changes, Apgar less than 7 at five minutes or maternal fevers [55].

#### Unfavorable cervix subgroup

There was no information about the efficacy of corticosteroids for induction of labor among women with unfavorable cervices [55].

*Summary: The use of corticosteroids for induction of labour is currently investigational*.

### Relaxin

Our search identified one Cochrane review that combined four studies including 267 women who used relaxin for induction of labour [56]. Compared with placebo or no treatment, relaxin reduced the number of participants with unfavourable cervices after 24 hours (3 studies, 173 women, 21/96 versus 37/75; RR 0.45, 95% CI 0.28 to 0.72; NNT = 4), but the need for oxytocin augmentation was not reduced (3 trials, 196 women, 65/121 versus 53/75; RR 0.83, 95% CI 0.65 to1.06). There were no differences in the rates of cesarean delivery, operative vaginal deliveries, or uterine hyperstimulation without FHR changes. There were insufficient data to evaluate perinatal death or morbidity [56].

#### Unfavorable cervix subgroup

In three trials with 207 women, there was no difference in caesarean deliveries in women allocated to receive relaxin compared with placebo [56].

*Summary: The use of relaxin for induction of labour is currently investigational*.

### Hyaluronidase

We identified one Cochrane systematic review that included one RCT with 168 women [57]. Women were randomly assigned to undergo intracervical hyaluronidase or placebo injections. Women receiving hyaluronidase injections were significantly less likely to require cesarean section (15/83 versus 42/85; RR 0.37, 95% CI 0.22 to 0.61; NNT = 4) and were less likely to require oxytocin augmentation (8/83 versus 40/85; RR 0.20, 95% CI 0.10 to 0.41; NNT = 3). Fewer women allocated to hyaluronidase had a cervix unfavorable/unchanged after 24 hours 50/83 versus 83/85, RR 0.62, 95% CI 0.52 to 0.74, NNT = 3), No adverse effects were reported [57].

#### Unfavorable cervix subgroup

This included systematic review did not report any subgroup analyses according to cervical status [57].

*Summary: The use of hyaluronidase for induction of labour is currently investigational*.

### Isosorbide Mononitrate

Our search identified two randomised controlled trials with a total of 502 participants comparing isosorbide mononitrate, a nitric oxide donor, with placebo [58] or PGE2 [59]. The Habib trial compared isosorbide mononitite tablets with a pyridoxine placebo; after receiving the trial medication, subjects received PGE2 or oxytocin according to hospital protocol. Compared with placebo, isosorbide mononitrate reduced the number of women who required treatment with PGE2 (32 of 51 versus 46 of 51, *P *= 0.002). However, isosorbide mononitrate increased the need for oxytocin augmentation (48 of 51 versus 27 of 51, *P *= 0.0001). More women who received placebo experienced uterine tachysystole. Compared with placebo, isosorbide mononitrate significantly shortened the admission to delivery interval (102 women, 13.45 +/- 6.63 versus 20.12 +/- 8.19; *P *= 0.0001). There was no difference in vaginal deliveries or cesarean sections between the groups [58]. The PRIM study compared isosorbide mononitrate to PGE2 [59]. Compared with PGE2, isosorbide mononitrite significantly lengthened the time from treatment to delivery (398 participants, 39,7 ± 12.0 hours versus 26.9 *+*.12.5 hours, mean difference -12.8 hours, 95% CI -15.2 hours-- -10.4 hours, *P *< 0.0001). There was no difference in spontaneous vaginal deliveries, operative vaginal deliveries, or cesarean sections [59].

#### Unfavorable cervix subgroup

Both the Osman and Habib trials required the qualifying women to have cervices unfavorable for induction [58,59].

*Summary: The use of isosorbide mononitrite for induction of labour is currently investigational*.

## Discussion

Our best-evidence review of the literature suggests that many commonly-recommended methods for induction of labour have important trade-offs between benefits and harms. Compared with placebo, use of vaginal and cervical prostaglandin E2 was consistently associated with reduced likelihood of failure to deliver vaginally within 24 hours but increased risk for hyperstimulation with and without FHR changes. Vaginal misoprostol reduced failure to achieve vaginal delivery within 24 hours compared with vaginal and cervical PGE2, but increased uterine contractile abnormalities. Likewise, vaginal misoprostol reduced caesarean deliveries compared with IV oxytocin, but increased uterine hyperstimulation. Mechanical methods for induction of labour were associated with reduced rates of uterine hyperstimulation compared with vaginal PGE2 and vaginal misoprostol, but were also associated with increased risk for maternal and neonatal infectious complications in the one included systematic review that compared mechanical methods with all other methods pooled. Intravenous oxytocin with and without amniotomy did not appear to have significant benefits compared with vaginal PGE2.

Of the non-pharmacologic methods, membrane sweeping appeared to have the strongest evidence-base. It was successful in reducing post-term gestations without increasing clinically-important harms. There is not enough evidence of benefit/safety to recommend the other non-pharmacologic methods of breast stimulation and sexual intercourse.

Our review included evaluation of several investigational methods of induction of labour, of which hyaluronidase appears the most promising. In one small trial, hyaluronidase reduced the need for oxytocin augmentation and for caesarean delivery. These findings need to be confirmed in large, appropriately-powered randomised controlled trials.

Our review may have been limited by restricting our search to the English-language literature and by publication bias. Because we used the Cochrane hierarchy, we compared each method of labour induction only with methods above them on the Cochrane hierarchy list. This may have limited the total number of comparisons made. Likewise, the included studies contained heterogeneous populations of women with both intact and ruptured membranes and cervices favourable and unfavourable for induction. The large number of methods of induction considered in our review precluded subgroup analyses according to membrane status. Likewise, we were not able to consider variation in pharmacologic preparation and dosing of the different compounds under study. In our review of methods of induction of labor in the setting of unfavorable cervix, we did not identify a clear best choice for induction of labor in this setting.

Despite the large amount of evidence that we were able to bring to bear on several common methods of labour induction, we also found considerable imprecision surrounding benefits and harms of many of the included methods. Numbers of included women in most induction randomized trials were too small to exclude differences in rare adverse outcomes such as uterine rupture, amniotic fluid embolism, or perinatal asphyxia. Further research is necessary to identify potential risks and benefits of both commonly-used and investigational methods of induction of labour.

## Conclusion

Clinicians should use the best available evidence to choose methods of labour induction. Researchers and funding agencies should prioritize studies that can help to definitively guide care in these situations. Women should be given information about what is known and not known regarding methods of induction in order to be able to participate fully in making decisions about induction of labour.

## List of Abbreviations

The abbreviations used in this manuscript include: CI: confidence interval; FHR: fetal heart rate; IV: intravenous; MA: meta-analysis; NNH: number needed to harm; NNT: number needed to treat; PGE2: Prostaglandin E2; PGF2α: Prostaglandin F2α; RCT: randomised controlled trial; RR: risk ratio; SR: systematic review; WMD: weighted mean difference.

## Competing interests

Drs. Mozurkewich, Romero, Berman, and Perni, are co-investigators on an ongoing multi-centre, industry-sponsored randomized controlled trial comparing the misoprostol vaginal insert with the dinoprostone vaginal insert.

## Authors' contributions

KK and JC performed the literature searches required for this review and reviewed all abstracts. EM and JC performed an updated search of the literature and abstract review when it became necessary during manuscript preparation. EM and KK reviewed all full text articles. EM and JC performed all assessments of study quality. EM, KK, DB, VR, UP, and VK wrote and edited the manuscript. KK and VK participated in the formulation of the methods of this review and EM and KK assigned the evidence grades. All authors read and approved the final manuscript.

## Pre-publication history

The pre-publication history for this paper can be accessed here:

http://www.biomedcentral.com/1471-2393/11/84/prepub
